# Human Adenovirus B3 Induced VITT

**DOI:** 10.1056/NEJMc2605960

**Published:** 2026-09-17

**Authors:** Christian Irsara, Habib Benainouna, Jing Jing Wang, Andrea Griesmacher, Christoph Winkler, Lorin Loacker, Markus Anliker, Xiaoyan Wang, Alex Moser, Thomas Müller, Werner Streif, Andreas Greinacher, Thomas Thiele, Wibke Bayer, Linda Schönborn

**Affiliations:** 1. Medical University of Innsbruck, Innsbruck, Austria; 2. Flinders University, Adelaide, SA, Australia; 3. University Hospital Essen, Essen, Germany; 4. Universitätsmedizin Greifswald, Greifswald, Germany

## To the Editor:

Virus-induced immune thrombocytopenia and thrombosis (VITT) occurs after adenoviral vector–based vaccination^1^ and natural adenovirus infection^2^. The underlying mechanism involves antibodies initially directed against the adenoviral core protein VII (pVII), which change their specificity towards platelet factor 4 (PF4) through somatic hypermutation and partial molecular mimicry^3^. The relevant epitope on pVII is encoded by a specific 15 amino acid sequence. However, it is unresolved which of the 15 amino acids are critical for antibody induction. The adenovirus vector platform is highly promising for developing affordable vaccines for neglected diseases and pandemic preparedness. Characterization of pVII sequence variants associated with postinfectious VITT may provide relevant information for future adenoviral vector redesign strategies aimed at reducing VITT risk.

We report a previously healthy 12-year-old boy who developed VITT approximately one week after onset of respiratory infection symptoms with severe thrombocytopenia (17,000 per cubic millimeter [155,000-425,000]), elevated D-dimer (12,852 ng per milliliter [<500]), hypofibrinogenemia (40 mg per deciliter [223-418]), and venous thromboses, including iliofemoral deep vein thrombosis and pulmonary embolism (Figure 1A). Laboratory testing showed strongly positive anti–PF4/heparin IgG by ELISA (optical density, 2.7; reference <0.3) and PF4-dependent platelet activating antibodies by PF4-induced platelet activation assay (PIPA). The causative anti-PF4 antibodies expressed the typical stereotypic VITT clonotype with a characteristic somatic K31E hypermutation on an IGLV3-21*02 light chain^3,4^. Adenovirus was isolated from a nasopharyngeal swab, and sequencing of a hexon gene fragment identified human adenovirus type 3 (species B; HAdV-B3). Serology confirmed specific anti-HAdV-B3 IgG and HAdV-B3–neutralizing antibodies already within one week of infection-related respiratory symptoms, along with anti-HAdV-B3 IgM, which persisted for eight months. The pVII protein of HAdV-B3 contains the amino acid sequence _93_AYARRKSRRRRIARR_107_, which differs slightly from the previously identified sequences of ChAdOx1 and HAdV-D26 (Figure 1B+C).

This first report of postinfectious VITT with sequencing of the causative adenovirus provides direct evidence for a shared, motif-driven mechanism linking natural human adenoviral infection and adenoviral vector–induced VITT. The early presence of HAdV-B3-neutralizing antibodies in the admission sample further supports that VITT represents a secondary immune response^3^. Finally, we show that clinicians should undertake all efforts to isolate the provoking adenovirus in newly diagnosed patients with VITT after natural adenovirus infection. A multidisciplinary effort integrating clinical, virological, and vaccine development expertise will be pivotal for isolating and sequencing the causative adenovirus, delineating VITT-relevant pVII determinants in vivo, and informing future adenoviral vector design strategies aimed at improving vaccine safety.

## Figures and Tables

**Figure 1.**
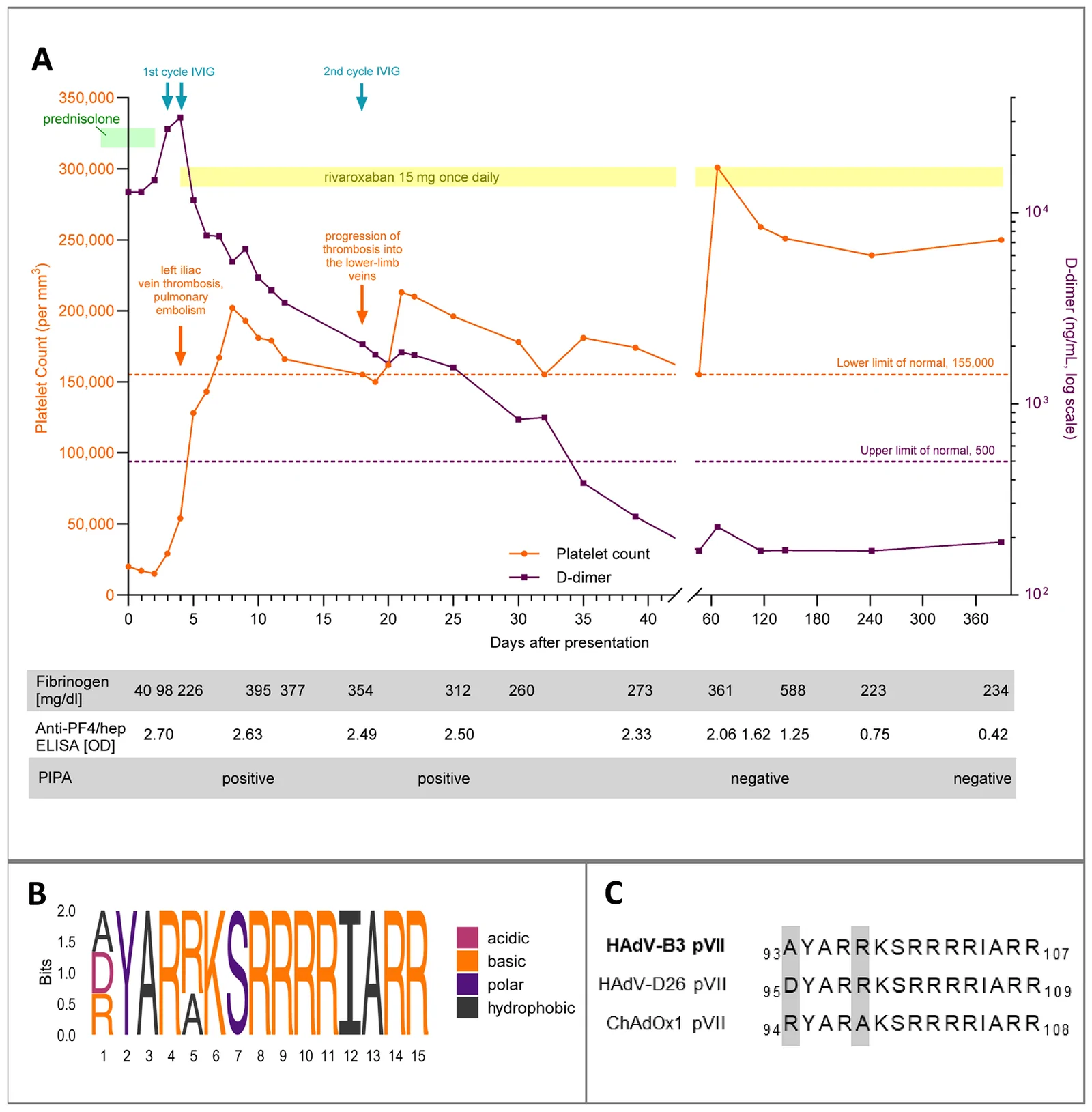
Clinical Course and pVII Sequence Analysis in VITT **A)** Clinical course of a previously healthy 12-year-old boy who presented with petechiae and a small hematoma on one arm approximately one week after onset of a respiratory adenovirus infection. Initially, immune thrombocytopenia (ITP) was suspected, and prednisolone treatment with 30 mg daily was started at the referring hospital. On admission to the university clinic, laboratory evaluation revealed severe thrombocytopenia (17,000 per cubic millimeter [155,000–425,000]), markedly elevated D-dimer levels (12,852 ng per milliliter [<500]), and hypofibrinogenemia (40 mg per deciliter [223–418]). On hospital day 2, strongly positive anti–PF4/heparin IgG antibodies were detected by ELISA (Hyphen BioMed; optical density, 2.7; cut-off ≥0.3), leading to the diagnosis of post-viral VITT, which was confirmed on day 9 by a positive PF4-enhanced platelet activation assay (PIPA). Further testing for hypercoagulability disorders identified a heterozygous factor V Leiden (R506Q) mutation. After an initially unremarkable ultrasound examination on hospital day 1, thrombosis of the left iliac veins and bilateral peripheral pulmonary emboli in the lower lobes were diagnosed on day 4. Prednisolone therapy was discontinued after a 4-day course (day 2 after admission), and treatment with intravenous immunoglobulin (IVIG; 1 g/kg body weight on two consecutive days) and rivaroxaban (15 mg daily) was initiated. This led to a rapid increase in platelet counts into the low-normal laboratory reference interval by day 6. However, thrombosis subsequently progressed into the lower-limb veins until day 18, prompting administration of a second IVIG cycle (2 g/kg body weight). Although platelet counts returned to the normal reference range soon after the first IVIG treatment, they remained substantially below the patient’s usual platelet count (>300,000/μL) and thrombosis progressed, suggesting that reaching the normal reference range does not always indicate full recovery for patients with VITT. At the most recent follow-up on day 389, the boy was asymptomatic, and ultrasound demonstrated a persistent thrombosis of the distal common iliac and external iliac veins, with partial central recanalization of the external iliac vein, while the common femoral vein had become patent. **B)** HAdV-B3 is a highly prevalent respiratory adenovirus associated with acute respiratory disease, particularly among children and adolescents. Differential display of the 15 amino acid sequence of pVII across different adenoviruses may help to narrow down clinically relevant residues involved in VITT antibody induction. Sequence logo of the alignment of the adenoviral core protein pVII 15mer epitope sequences of HAdV-B3, HAdV-D26, and ChAdOx1. **C)** The individual epitope sequences with the divergent amino acids highlighted in grey. The three adenoviruses which are known to induce VITT in vivo in humans differ in amino acid 1 and 5 of the 15mer epitope sequence. This information may help guide strategies to modify pVII in next generation adenovirus vectors to reduce the risk of VITT. Protein sequences were obtained from the NCBI database, accession numbers: KX384958.1 (HAdV-B3), EF153474.1 (HAdV-D26), NC_017825 (ChAdY25). Sequence logo created using R ggseqlogo package.

## Abbreviations:

ELISA: enzyme-linked immunosorbent assay
IVIG: intravenous immunoglobulins
OD: optical density
PF4: platelet factor 4
PIPA: PF4-enhanced platelet activation assay
